# Supplementary material for: Atrazine Desorption Mechanism from an Hydrated Calcium Montmorillonite—A DFT Molecular Dynamics Study
Source: Int J Mol Sci. 2024 Jan 27;25(3):1604. doi: 10.3390/ijms25031604 (PMC10855099; doi:10.3390/ijms25031604)
Supplement: Supplementary file 1 [file ijms-25-01604-s001.zip › ijms-2812314-supplementary.pdf]

Supplementary Materials: Atrazine Desorption Mechanism from an Hydrated Calcium Montmorillonite—A DFT Molecular Dynamics Study

Quentin Desdion [1,2], Fabienne Bessac [1,2,*] and Sophie Hoyau [1]

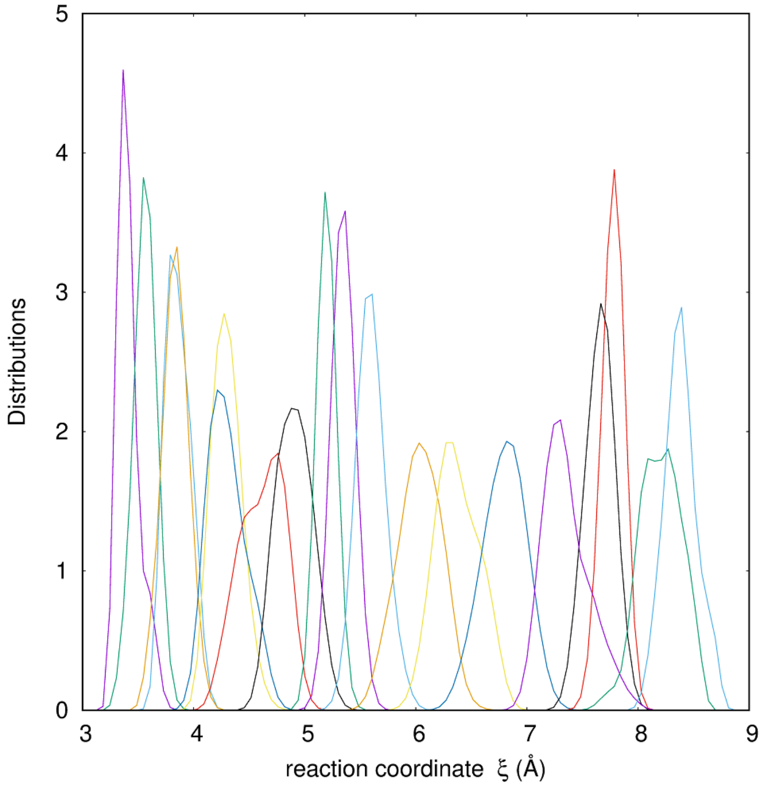

Figure S1. Overlapping distributions of ξ, the distance from the center of mass of atrazine to surf, obtained for the umbrella sampling simulations to determine the free energy profile of atrazine desorption from the montmorillonite surface (see Figure 3).

For all the trajectories, Oe–Oe and Oe–Ob radial distribution functions (RDF) were plotted to confirm the liquid phase of water all along the desorption. On Figure S2, the Oe–Oe and Oe–Ob RDF are given for each singularity:

- Oe–Oe distances between the oxygen atoms of water molecules – For each singularity, the profiles are almost stackable. In addition, the first peak of each distribution corresponds to a distance of $\approx$2.8 Å, which is characteristic of liquid water;
- Oe–Ob distances between the oxygen atoms of the water molecules Oe and the oxygen atoms of the surface Ob – Similarly, for each singularity, the distributions are comparable, which indicates a similar hydration of the surface all along the desorption. The first peak at $\approx$3.0 Å indicates that the water molecules are not in direct contact with the surface of the montmorillonite.

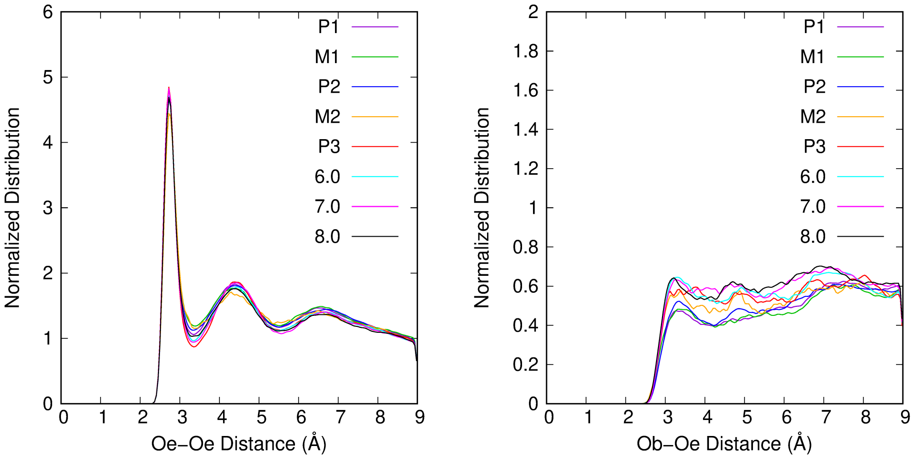

(**a**) (**b**)

Figure S2. Oe–Oe (**a**) and Ob–Oe (**b**) radial distribution functions for the singularities P1, M1, P2, M2 and P3 and for around the ξ values 6.0, 7.0 and 8.0 Å of the free energy profile of desorption of Fen from Mont. Oe are the oxygen atoms of water molecules and Ob, the surface oxygen atoms.

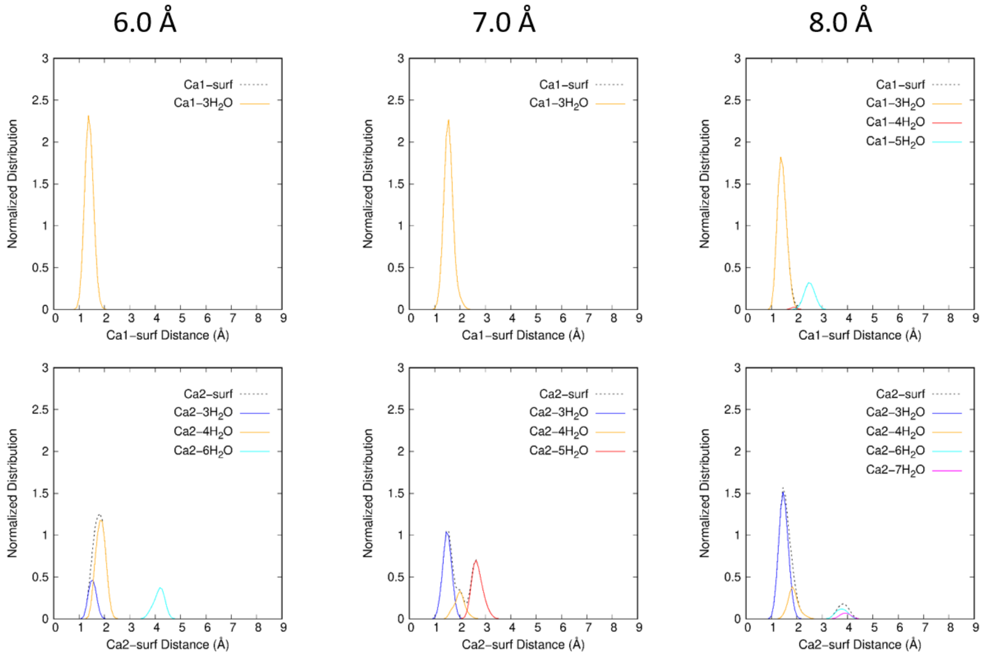

Figure S3. Ca1–surf and Ca2–surf normalized distributions for around the ξ values 6.0, 7.0 and 8.0 Å of the free energy profile. The structures were sorted according to the number of water molecules in the first HS of Ca1 or Ca2 ($d_{Oe-Ca} \leq 3.0$ Å).

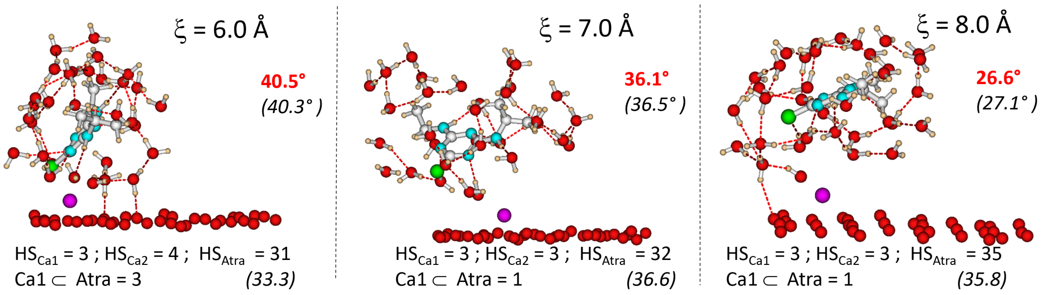

Figure S4. Snapshots taken for zones around the ξ values 6.0, 7.0 and 8.0 Å of the free energy profile and their corresponding ξ values. For a sake of clarity, only the Ob oxygen atoms are represented for the Mont surface. For the same reason, only the water molecules involved in the first HS of both atrazine and Ca1 are represented. HS_{Ca1}, HS_{Ca2} and HS_{Atra} the number of water molecules involved in the first HS of each entity are noticed. In red, the θ angle is given for the snapshot. In parentheses, the mean values of HS_{Atra} and θ are also given.

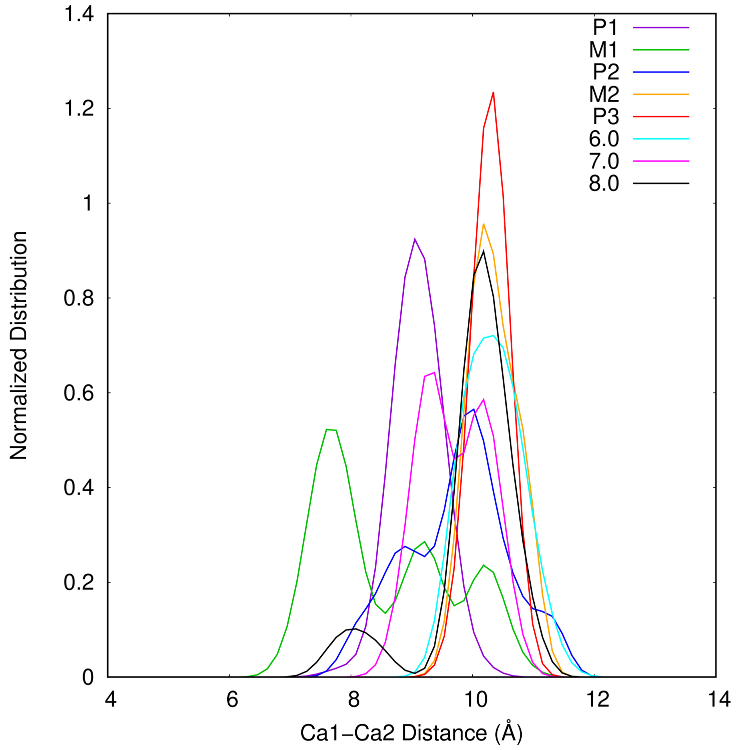

Figure S5. Ca1–Ca2 normalized distributions for the singularities P1, M1, P2, M2 and P3 and for around the ξ values 6.0, 7.0 and 8.0 Å of the free energy profile.

Table S1. The number of structures, N, found in each singularity P1, M1, P2, M2 and P3 and also for around the ξ values 6.0, 7.0 and 8.0 Å are given.

	N	HS_{Ca1}	HS_{Ca2}
**P1**	8209		
6341[a]	3684	1	4
	2657	1	5
1868	1085	2	4
	418	2	5
	365	2	6
**M1**	3936		
915	221	0	2
	302	0	3
	392	0	4
2847	438	1	4
	2262	1	5
	147	1	6
174	143	2	4
	10	2	5
	21	2	6
**P2**	6645		
299	114	0	5
	27	0	6
	158	0	7
3116	380	1	3
	235	1	4
	177	1	5
	1728	1	6
	596	1	7
3230	395	2	3
	675	2	4
	1678	2	5
	350	2	6
	132	2	7
**M2**	610		
82	36	0	1
	46	0	2
156	85	1	2
	60	1	3
	11	1	4
372	372	3	3
**P3**	2833		
2833	2833	3	3
**6.0 Å**	3604		
3604	682	3	3
	2201	3	4
	721	3	6
**7.0 Å**	3230		
3230	1497	3	3
	526	3	4
	1207	3	5
**8.0 Å**	3392		
2797	2402	3	3
	28	3	4
	243	3	6
	124	3	7
26	26	4	4
569	569	5	4

[a] Number of structures for each HS_{Ca1}.
